# Genetic Defects in Mitochondrial Dynamics in *Caenorhabditis elegans* Impact Ultraviolet C Radiation- and 6-hydroxydopamine-Induced Neurodegeneration

**DOI:** 10.3390/ijms20133202

**Published:** 2019-06-29

**Authors:** Jessica H. Hartman, Claudia Gonzalez-Hunt, Samantha M. Hall, Ian T. Ryde, Kim A. Caldwell, Guy A. Caldwell, Joel N. Meyer

**Affiliations:** 1 Nicholas School of the Environment, Duke University, Durham, NC 27708, USA; 2 Department of Biological Sciences, The University of Alabama, Tuscaloosa, AL 35487, USA

**Keywords:** mitochondrial fusion, mitochondrial fission, mitophagy, dopaminergic neurodegeneration, 6-hydroxydopamine, mitochondrial DNA damage

## Abstract

Background: Parkinson’s disease (PD) is one of the most common neurodegenerative disorders involving devastating loss of dopaminergic neurons in the substantia nigra. Early steps in PD pathogenesis include mitochondrial dysfunction, and mutations in mitochondrial genes have been linked to familial forms of the disease. However, low penetrance of mutations indicates a likely important role for environmental factors in PD risk through gene by environment interactions. Herein, we study how genetic deficiencies in mitochondrial dynamics processes including fission, fusion, and mitophagy interact with environmental exposures to impact neurodegeneration. Methods: We utilized the powerful model organism *Caenorhabditis elegans* to study ultraviolet C radiation (UVC)- and 6-hydroxydopamine-induced degeneration of fluorescently-tagged dopaminergic neurons in the background of fusion deficiency (MFN1/2 homolog, *fzo-1*), fission deficiency (DMN1L homolog, *drp-1*), and mitochondria-specific autophagy (mitophagy) deficiency (PINK1 and PRKN homologs, *pink-1* and *pdr-1*). Results: Overall, we found that deficiency in either mitochondrial fusion or fission sensitizes nematodes to UVC exposure (used to model common environmental pollutants) but protects from 6-hydroxydopamine-induced neurodegeneration. By contrast, mitophagy deficiency makes animals more sensitive to these stressors with an interesting exception—*pink-1* deficiency conferred remarkable protection from 6-hydroxydopamine. We found that this protection could not be explained by compensatory antioxidant gene expression in *pink-1* mutants or by differences in mitochondrial morphology. Conclusions: Together, our results support a strong role for gene by environment interactions in driving dopaminergic neurodegeneration and suggest that genetic deficiency in mitochondrial processes can have complex effects on neurodegeneration.

## 1. Introduction

Parkinson’s disease (PD) is a neurodegenerative disorder characterized by progressive loss of dopaminergic neurons in the substantia nigra. Clinical symptoms leading to diagnosis do not emerge until 50–70% of the dopaminergic neurons are lost, which is already late in the progression of the pathology. Genetic causes, particularly for early onset familial PD, account for roughly 5–10% of all cases of PD. The remaining majority of sporadic PD cases have unknown causes, but environmental exposures are expected to play a significant role [1,2]. Environmental toxicants may be especially relevant for more susceptible genetic backgrounds; gene by environment interactions could explain why an exposure only triggers PD in a subset of the exposed population [3,4]. The same may be true of Parkinsonism and other dopaminergic neurodegeneration-related pathologies.

One important mechanism through which genes and environment may interact is at the level of mitochondrial dysfunction [5,6]. PD pathogenesis involves mitochondrial dysfunction as a key initiating step in the neuronal dysfunction and death [7]. Additionally, many studies have utilized rotenone, a specific mitochondrial complex I inhibitor, to target dopaminergic neurons and model PD [5]. Most other chemicals strongly associated experimentally, epidemiologically, and/or clinically with PD or parkinsonism are also mitochondrial toxicants, including 1-methyl-4-phenyl-1,2,3,6-tetrahydropyridine (MPTP), paraquat, manganese, and trichloroethylene. Genetic disruption of mitochondrial function also leads to dopaminergic neurodegeneration, including a mouse model with the mitochondrial transcription factor TFAM selectively silenced in dopaminergic neurons that shows progressive induction of mitochondrial dysfunction and nigral neuronal cell death as the animals reach early adulthood [8]. Additionally, mice lacking mitophagy-related Parkin in combination with a “mutator” form of proofreading-deficient polymerase gamma (responsible for replication of mitochondrial DNA, or mtDNA) also undergo early loss of dopaminergic neurons in the substantia nigra [9].

Further evidence for mitochondrial involvement in PD pathogenesis comes from human genetic studies. The genes associated with familial PD include mutations in genes involved in mitophagy (*PINK1* and *PRKN)*, a special form of autophagy that is selective for damaged mitochondria [10]. Many other PD genes also have mitochondria-related functions or cause detrimental mitochondrial phenotypes when mutated [5,11,12,13,14,15]. Interestingly, although *PINK1* and *PRKN* mutations are autosomal recessive, there is some controversial evidence that heterozygous carriers of *PINK1* and *PRKN* mutations may be more susceptible, potentially through environmental contributions [16,17,18]. Furthermore, the role of the environment in the age-of-onset and the disease progression in the background of heterozygous or homozygous *PINK1* and *PRKN* mutations is not well understood.

**Figure 1:**
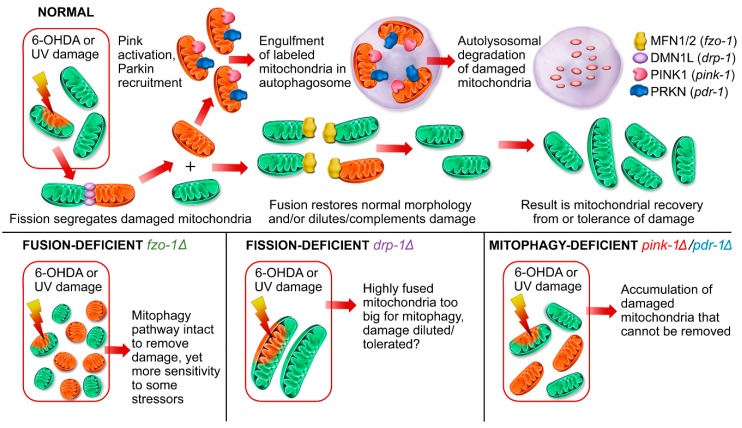
A role for mitochondrial dynamics in removing damage from 6-hydroxydopamine (6-OHDA) or ultraviolet C radiation (UVC) radiation. In this study, we hypothesize that genetic alterations in mitochondrial dynamics processes diminish the ability of dopaminergic neurons to respond to mitochondrial damage caused by UVC radiation or 6-OHDA exposure. Orange mitochondria represent damaged organelles, while green mitochondria are undamaged. The protein legend in the top right of the figure provides the human protein names (e.g., MFN1/2) with the corresponding *Caenorhabditis elegans* homolog (e.g., *fzo-1*) in parentheses. Genetic mutations (indicated by a Δ symbol) in this figure are also depicted using the same colors in Figure 2, Figure 3, Figure 4 and Figure 5.

In addition to mitophagy, mutations in other genes involved in mitochondrial dynamics may also interact with environmental exposures to initiate or drive mitochondrial toxicity, as we have recently reviewed [19], and contribute to PD initiation or progression. These processes include mitochondrial fusion—or the combination of two mitochondria to form a larger organelle—and mitochondrial fission, which is the segregation of one mitochondrion into two daughter mitochondria [19]. Deficiencies in these processes can lead to mitochondrial disease, including Charcot-Marie Tooth neuropathy in people carrying a mutation in MFN2, a gene necessary for mitochondrial fusion [20]. Supporting the likelihood that these sensitivities are relevant in the context of dopaminergic neurodegeneration, there is evidence for an important role for MFN2 in dopaminergic neuron health [21], and there are direct interactions between the PINK, MFN2, and Parkin [22]. Even in the absence of symptomatic disease, changes to mitochondrial dynamics can change the mitochondrial morphological landscape and may influence how mitochondria respond to damage, as depicted in Figure 1. 

In this study, we investigate the role of genetic deficiencies in mitodynamic processes in promoting neurodegeneration caused by exposure to mtDNA-damaging ultraviolet C radiation (UVC) radiation and by exposure to the model neurotoxicant 6-hydroxydopamine (6-OHDA). We previously documented dopaminergic neurodegeneration from such exposures in wild-type animals [23]. We employed UVC radiation as a model for environmental and food pollutants as well as certain drugs. UVC itself does not penetrate the stratospheric ozone. Ultraviolet B radiation (UVB) does and can cause some similar kinds of DNA damage (photodimers), but neither penetrates deeply into the human body. However, because UVC-induced photodimers in mtDNA are irreparable due to the absence of nucleotide excision repair in the mitochondria, they serve as a model for the kinds of “bulky,” helix-distorting damage caused by very common environmental pollutants such as many polycyclic aromatic hydrocarbons found in polluted air, food genotoxicants such as heterocyclic amines and mycotoxins, and drugs such as cisplatin [3]. Herein, we utilize strains deficient in mitochondrial dynamic processes (fusion, fission, and mitophagy) crossed into a transgenic reporter GFP driven by the promoter of the *dat-1* dopamine transporter to visualize direct damage to dopaminergic neurons as previously described [23,24]. Using this method, we report for the first time interactions of genetic mutations in mitochondrial dynamics with UVC- and 6-OHDA-induced neurodegeneration. Genetic deficiencies in mitochondrial fission and especially mitochondrial fusion sensitized animals to UVC exposure, but we observed remarkable protection from 6-OHDA-induced neurodegeneration in the background of mutations in mitochondrial fusion and fission. Conversely, mutations in mitophagy genes generally led to sensitization of neurons to damage. A fascinating exception to that trend was remarkable protection of *pink-1* mutants from 6-OHDA-induced neurodegeneration. We tested two hypotheses for the mechanism underlying this protection, including differential expression of antioxidant genes and changes to mitochondrial morphology. Together, these results give new insight into gene–environment interactions in neurodegeneration when genetic mutations that perturb mitochondrial dynamics are present. 

## 2. Results

### 2.1. Genetic Deficiencies in Mitochondrial Fission and Fusion Impact Environmentally-Induced Neurodegeneration

Mitochondrial dynamics are critical to maintaining normal mitochondrial homeostasis and for responding to damage from environmental insults. We previously reported a requirement for mitochondrial fusion and fission in removing mtDNA lesions caused by UVC damage [25]. Furthermore, we found that deficiencies in mitochondrial fission and fusion altered mitochondrial morphology and function [26] and sensitized nematodes to toxicity (growth delay) from arsenite and other mitotoxicants [27]. Therefore, we assessed whether nematodes deficient in mitochondrial fusion or fission would be sensitized to UVC damage-induced dopaminergic neurodegeneration compared to wild-type animals. 

#### 2.1.1. Mitochondrial Fusion Deficiency Sensitized Nematodes to UVC-Induced L1 Larval Arrest and Lethality

**Figure 2:**
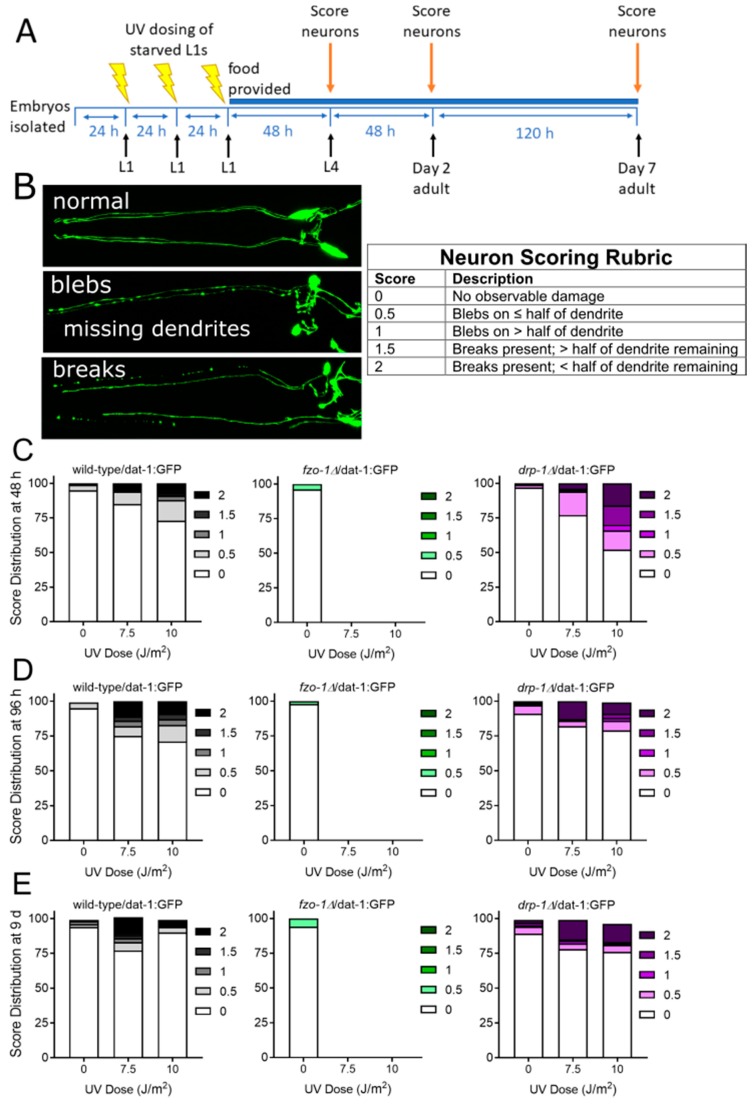
Genetic deficiencies in mitochondrial fusion cause UVC-induced L1 arrest and lethality in fusion-deficient *fzo-1Δ* worms, and fission-deficient *drp-1Δ* worms are sensitized to UVC-induced neurodegeneration. For experiments, synchronized L1 larvae were exposed to UVC radiation on three consecutive days and then provided with food and allowed to develop (Panel **A**). Neurodegeneration (as shown in Panel **B**) was then measured 48 h (Panel **C**), 96 h (Panel **D**), and 9 days (Panel **E**) following exposure. Scoring, as shown in Panel B, was performed as described in Methods: 0 represents no degeneration, while increasing numbers represent increasing severity of degeneration. Data shown are combined from three biological replicates with each treatment group comprising *n* > 40 worms scored for dendrites on each of 4 cephalic neurons for a total of >120 dendrites scored.

Cells lacking the capacity to fuse mitochondria have naturally fragmented mitochondria at baseline and are unable to rescue dysfunctional mitochondria via fusion-enabled functional complementation. However, fusion-deficient cells can remove dysfunctional mitochondria through canonical mitophagy pathways (Figure 1). We hypothesized that nematodes deficient in mitochondrial fusion (*fzo-1Δ*) would be more susceptible to dopaminergic neurodegeneration compared to wild-type animals following UVC exposure. We used a UVC exposure protocol (Figure 2A) that results in a high level of accumulated mitochondrial DNA (mtDNA) damage while permitting repair of detectable nuclear DNA damage [28] and causes mild dopaminergic neurodegeneration [23]. Interestingly, the UVC exposure caused complete L1 arrest and death in *fzo-1* mutant animals, which unfortunately prevented us from assessing the impact of the UVC exposure on neurodegeneration.

#### 2.1.2. Deficiency in Mitochondrial Fission Exacerbates UVC-Induced Neurodegeneration

Mitochondrial fission (division of mitochondria) was identified as an important step in removing damaged cargo in mitochondria, as illustrated in Figure 1. Therefore, we hypothesized that fission-deficient *drp-1* mutant animals would be more sensitive to neurodegeneration from exposure to UVC radiation. We found that 48 h following exposure, *drp-1* mutant worms had significantly more neurodegeneration compared to wild-type at the highest dose (Figure 2B, Table 1). However, after 48 h of additional recovery, the *drp-1* mutants overcame their sensitivity and showed some protection compared to wild-type animals at the 7.5 J/m^2^ dose (Figure 2C). Finally, we looked at older animals nine days following exposure. Interestingly, *drp-1* mutants trended toward more neurodegeneration from aging (*p* = 0.1157) and significantly worse neuronal integrity after the highest dose of UVC exposure (Figure 2D). Together, these results suggest that, in general, *drp-1* depletion appears to mildly sensitize animals to UVC-induced neurodegeneration. 

#### 2.1.3. Mitochondrial Fission and Fusion Mutants are Protected from 6-OHDA-Induced Neurodegeneration

**Figure 3:**
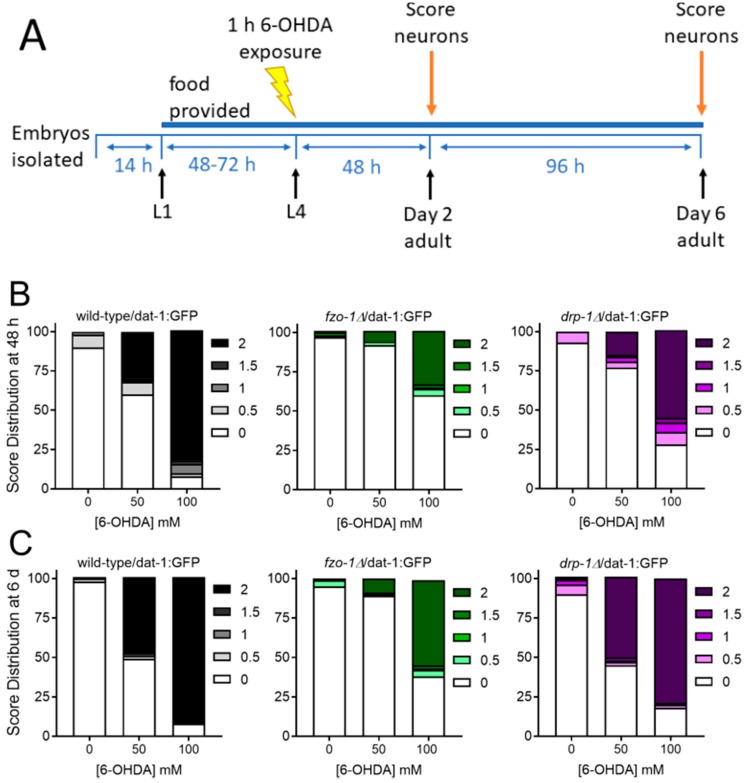
Worms deficient in mitochondrial fission and fusion are protected from 6-OHDA-induced neurodegeneration. For experiments, synchronized L1 larvae were provided with food and allowed to develop to the L4 stage, which was 48 h in wild-type and *drp-1* mutants and 72 h in slow-growing *fzo-1* mutants (Panel **A**). L4 worms were then exposed to 6-OHDA for 1 h, and neurodegeneration was measured 48 h (Panel **B**) and six days (Panel **C**) following exposure. Scoring was performed as described in Methods: 0 represents no degeneration, while increasing numbers represent increasing severity of degeneration. Data shown are combined from three biological replicates with each treatment group comprising *n* > 40 worms scored for dendrites on each of 4 cephalic neurons for a total of >120 dendrites scored.

6-OHDA is a neurotoxicant that particularly targets dopaminergic neurons because it is a substrate for the dopamine transporter and is believed to act by causing oxidative damage. It also causes significant mtDNA damage but causes a much higher degree of dopaminergic neurodegeneration than does UVC [23], perhaps because it targets a wider range of macromolecules than UVC, which primarily damages nucleic acids. We employed this prototypical dopaminergic neurotoxicant to compare environmental toxicant-induced dopaminergic neurodegeneration in wild-type animals and those deficient in mitochondrial fusion and fission. L4 larval nematodes were exposed to 6-OHDA and scored 48 h and six days later (Figure 3A). Each strain showed a dose-dependent increase in neurodegeneration. Interestingly, there were very few intermediate scores after 6-OHDA exposure; neurons were either intact (score 0) or highly degenerated (scores 1.5 and 2). Compared to the wild-type strain, both *fzo-1* and *drp-1* mutants were protected from 6-OHDA toxicity 48 h after exposure (Figure 3B, statistics in Table 2), with the protection being most dramatic in the *fzo-1* mutant. This protection was maintained even six days following exposure (Figure 3C, statistics in Table 2). 

### 2.2. Animals Lacking Classic Pink1- and Parkin-Mediated Mitophagy are Generally more Sensitive to UVC- and 6-OHDA-Induced Neurodegeneration

Mitophagy is a major pathway by which mitochondrial damage is cleared away (Figure 1), and mutations in mitophagy-related genes *PINK1* and *PRKN* have been linked to familial PD in humans. We previously reported that *Caenorhabditis elegans* deficient in PINK1 (*pink-1)* and PRKN (*pdr-1*) are more sensitive to growth delay from exposure to mitochondrial toxicants, including rotenone and 2,4-dinitrophenol [27]. Therefore, we wanted to test whether genetic deficiencies in these genes would cause higher neurodegeneration in the presence of UVC radiation or 6-OHDA. 

#### 2.2.1. Exposure to UVC Caused more Neurodegeneration when Mitophagy was Blocked in *pink-1* or *pdr-1* Mutants

**Figure 4:**
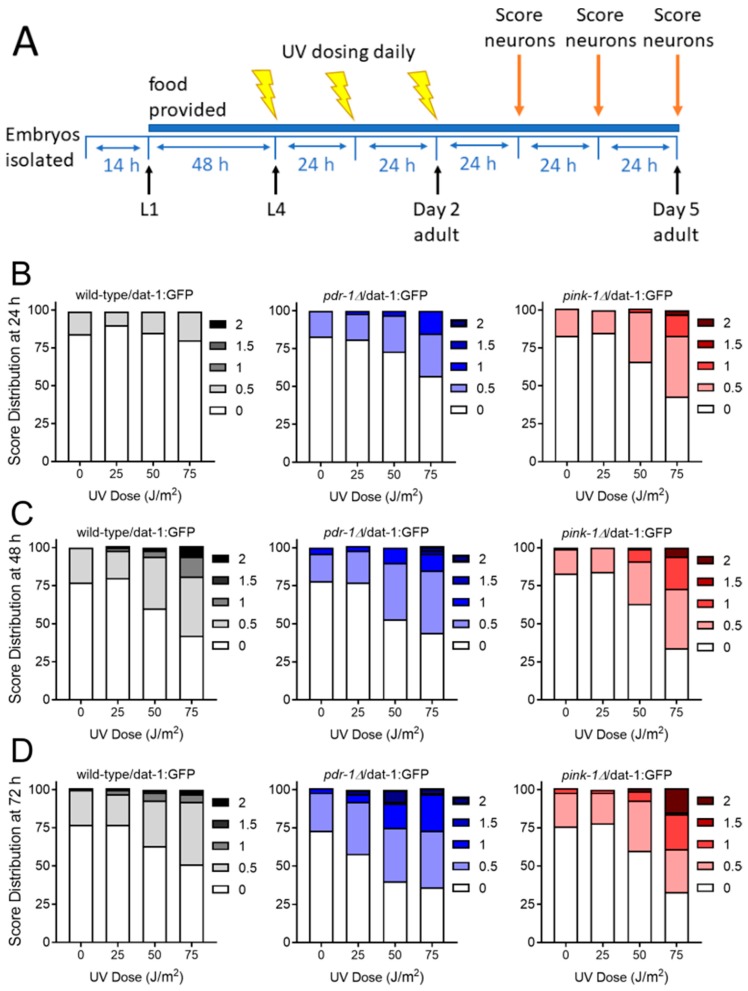
Genetic deficiencies in mitophagy sensitize worms to UVC-induced neurodegeneration. For experiments, wild-type, *pink-1* mutant, and *pdr-1* mutant worms were exposed to UVC radiation on three consecutive days beginning at the L4 larval stage (Panel **A**). Neurodegeneration was then measured 24 h (Panel **B**), 48 h (Panel **C**), and 72 h (Panel **D**) following exposure. Scoring was performed as described in Methods: 0 represents no degeneration, while increasing numbers represent increasing severity of degeneration. Data shown are combined from three biological replicates with each treatment group comprising *n* > 30 worms scored for dendrites on each of 4 cephalic neurons for a total of >120 dendrites scored.

UVC-induced DNA damage is repaired quickly in nuclear DNA, but mitochondrial damage persists much longer due to the absence of nucleotide excision repair [25]. We previously reported that the slow removal of damaged mtDNA that does occur is slowed by *pink-1* mutation [25]. Therefore, we wondered if mutants deficient in mitophagy might be particularly susceptible to UVC-induced neurodegeneration. We exposed animals on three consecutive days to UVC exposure and measured neurodegeneration on the following three days (Figure 4A). We found that even 24 h after exposure, there was increased neurodegeneration only in the *pink-1* and *pdr-1* mutants with no increased damage in the wild-type exposed animals (Figure 4B, Table 3). Over time, some neurodegeneration emerged in the wild-type animals (Figure 4C,D); however, the damage was more significant in the mitophagy-deficient mutants, especially on the last day (Table 3). 

#### 2.2.2. Mutants Deficient in *pdr-1* Are More Sensitive to 6-OHDA-Induced Neurodegeneration, but *pink-1* Mutants Are Protected

**Figure 5:**
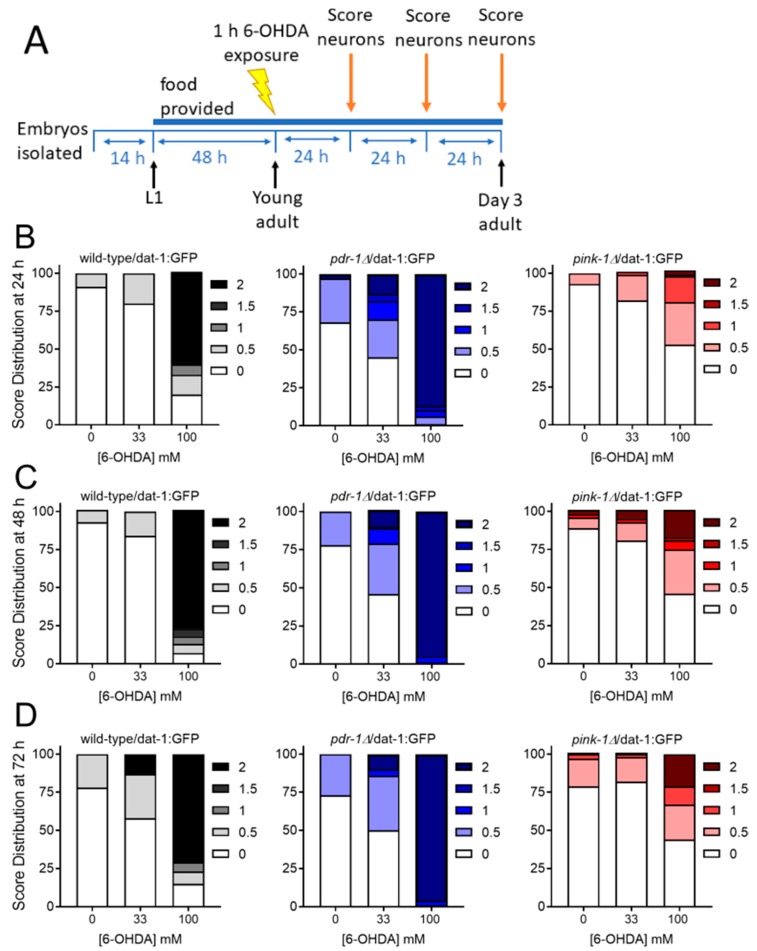
Loss of *pdr-1* sensitizes nematodes to 6-OHDA, but loss of *pink-1* is protective against 6-OHDA. For experiments, wild-type, *pink-1* mutant, and *pdr-1* mutant worms were exposed to 6-OHDA for one hour as young adults (Panel **A**). Neurodegeneration was then measured 24 h (Panel **B**), 48 h (Panel **C**), and 72 h (Panel **D**) following exposure. Scoring was performed as described in Methods: 0 represents no degeneration, while increasing numbers represent increasing severity of degeneration. Data shown are combined from three biological replicates with each treatment group comprising *n* > 30 worms scored for dendrites on each of 4 cephalic neurons for a total of >120 dendrites scored.

As mentioned, 6-OHDA causes relatively specific toxicity in dopaminergic neurons, and this toxicity involves mitochondrial dysfunction. We hypothesized that impaired ability to remove such damage would sensitize animals to 6-OHDA toxicity. To test this, we exposed young adult nematodes to 6-OHDA for 1 h and monitored neurodegeneration in the three days following exposure (Figure 5A). In support of our hypothesis, *pdr-1* mutants were highly sensitive to 6-OHDA-induced neurodegeneration, which was apparent at both doses and all time points (Figure 5B–D, statistics in Table 4). However, to our surprise, *pink-1* mutants were protected from 6-OHDA-induced neurodegeneration. At 24 h following exposure at the highest dose, more than 60% of wild-type neurons and more than 80% of *pdr-1Δ* neurons showed severe damage (score 2), while <5% of *pink-1Δ* neurons demonstrated this severe phenotype (Figure 5B). Although neurodegeneration increased over the following days, remarkable protection in *pink-1* mutants was maintained 48 and 72 h following exposure (Figure 5C,D). 

### 2.3. Resistance to 6-OHDA-Mediated Dopaminergic Neurodegeneration in pink-1 Mutants Is not Mediated by Increased Antioxidant Gene Expression but may Involve Mitochondrial morphology Differences

#### 2.3.1. Pink-1 Mutants do not Differentially Express Antioxidant Genes at Baseline or in Response to 6-OHDA

**Figure 6:**
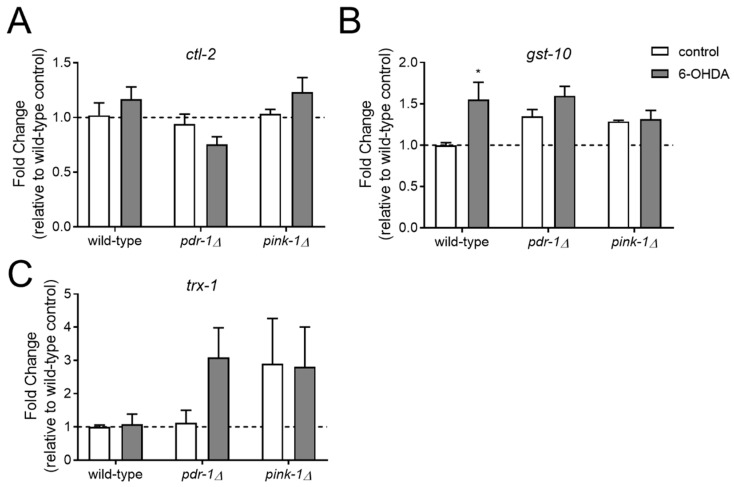
Antioxidant gene expression is not significantly altered in *pink-1* and *pdr-1* mutants. For experiments, wild-type, *pink-1* mutant, and *pdr-1* mutant worms expressing the *P_dat-1_::*GFP transgene were exposed to 6-OHDA (grey bars) or solvent (white bars) for one hour as young adults (similar to the exposure scheme shown in Figure 5, Panel **A**). RNA was isolated 24 h following exposure, and mRNA of catalase (*ctl-2*, panel A), glutathione S-transferase (*gst-10*, panel B), and thioredoxin (*trx-1,* panel **C**) was measured using qPCR as described in Methods. Asterisks represent significance (*p* < 0.05) compared to the non-exposed control of the same strain in a two-way ANOVA with Sidak’s multiple comparison test.

The results of the neurodegeneration experiments showed that, although the *pdr-1* mutant is more sensitive to both UVC- and 6-OHDA-induced neurodegeneration compared to wild-type, the *pink-1* mutant only showed increased sensitivity to UVC and was protected from 6-OHDA. We hypothesized that the *pink-1* mutant may have adapted to the loss of a functional mitophagy pathway by upregulating antioxidant stress response genes. This adaptation would potentially provide protection from 6-OHDA-induced mitochondrial dysfunction (which is thought to work through oxidative damage) but not the UVC-induced mtDNA damage. To test this, we measured mRNA expression for the antioxidant genes *ctl-2* (ortholog of human catalase), *gst-10* (ortholog of human glutathione S-transferase pi 1), and *trx-1* (ortholog of human thioredoxin). Only *gst-10* was upregulated in response to 6-OHDA exposure in the wild-type background, and no change was observed in the mitophagy deficient strains (Figure 6). In the absence of 6-OHDA, there was no significant difference between strains in antioxidant gene expression. 

#### 2.3.2. *pink-1* Mutant Mitochondrial Morphology Cannot Explain Differential Sensitivity to 6-OHDA

**Figure 7:**
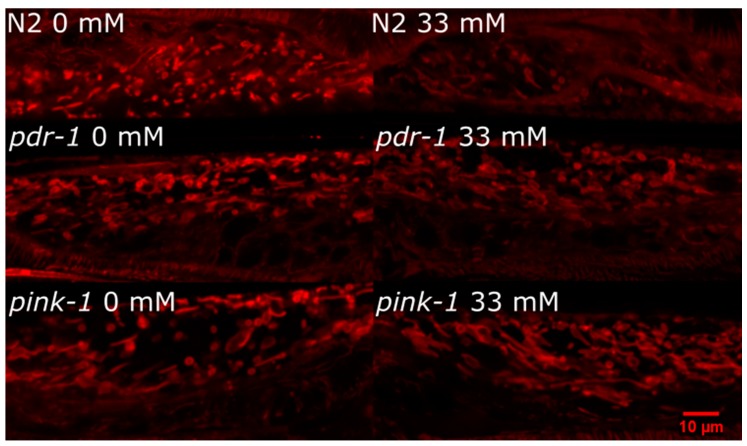
Mitochondrial morphology is not dramatically altered in *pink-1* or *pdr-1* mutants after 6-OHDA exposure compared to N2 (wild-type) animals. For experiments, worms (wild-type and mitophagy-deficient mutants crossed into the BY200 transgenic reporter strain) were exposed to 6-OHDA as in other experiments. Following exposure, mitochondrial morphology was visualized using MitoTracker CMXRos (incubated with worms for final 4 h of 24 h recovery after exposure). Confocal imaging was performed on animals immobilized with tetramisole and mounted on an agarose pad as described in Methods.

We previously reported that *pink-1* and *pdr-1* mutants increased fusion of mitochondrial networks in body wall muscle cells [26]. Because only *pink-1* but not *pdr-1* mutants showed resistance to 6-OHDA-induced neurodegeneration, we hypothesized that *pink-1* mutants would have a different response to 6-OHDA that could be visualized in the morphology of the mitochondrial networks. However, when we visualized the mitochondrial networks in each strain, we did not see an obvious difference between strains after 6-OHDA exposure (Figure 7). 

## 3. Discussion

In this study, we show that genetic deficiencies in mitochondrial dynamics have a dramatic effect on the sensitivity of *C. elegans* to environmentally-induced dopaminergic neurodegeneration. Our initial hypothesis that all deficiencies would sensitize, since fusion, fission, and mitophagy are all homeostatic processes, was incorrect. *drp-1* deficiency did indeed mildly sensitize to UVC-induced neurodegeneration, and fusion deficiency dramatically sensitized to UVC-induced lethality. However, we were surprised to find that deficiencies in mitochondrial fission and fusion were protective against 6-OHDA-induced neurodegeneration. Deficiencies in mitophagy tended to sensitize animals to neurodegeneration, as we expected. A fascinating exception to the generalization regarding mitophagy was the remarkable protection from 6-OHDA-induced neurodegeneration observed in *pink-1* mutant animals, which could not be explained by any tested antioxidant gene expression or overt mitochondrial morphological changes. A limitation of these experiments is that the mRNA expression levels were measured only at a single timepoint following exposure—changes at the protein level or dynamic mRNA expression changes at different timepoints cannot be completely ruled out. Nonetheless, the interaction of mitodynamic genes and mitotoxic exposures does not appear to be a simple story of sensitization due to genetic deficiency in all cases, as we initially hypothesized. Rather, as discussed below, our results as well as research from other groups in *C. elegans* and other model systems demonstrate that these mitochondrial gene–environment interactions are not as simple as predicted and require further study.

We and others reported previously that deficiency in fusion and fission proteins sensitizes animals to growth delay and other toxicity measurements after exposure to many environmental contaminants [19,27,29,30]. Consistent with this, we saw very dramatic lethality in *fzo-1* fusion-deficient mutant animals after UVC and sensitization to UVC-induced neurodegeneration in *drp-1* fission-deficient mutants. However, there are also examples where deficiency in these processes is associated with protection, which is what we observed in this study for both fusion and fission deficiency with the mitotoxicant 6-OHDA. Our fission results appear to be consistent with previous literature; exposure to 6-OHDA leads to increased mitochondrial fragmentation [31,32] and interventions that increase mitochondrial fusion generally protect from 6-OHDA-induced neurotoxicity [33,34]. In mice, genetic DRP1 deficiency and pharmacological inhibition of DRP1 protected against dopaminergic neurodegeneration caused by 1-methyl-4-phenyl-1,2,3,6-tetrahydropyridine (MPTP) and mutant α-synuclein expression [35,36]. A previous report in *C. elegans* showed that knock-down of both *drp-1* and *fzo-1* led to increased basal dopaminergic neurodegeneration, but protection against degeneration induced by a bacterial metabolite [37]. 

In mice, there is a progressive, age-dependent loss of dopaminergic neurons in animals deficient in the mitochondrial fusion protein MFN2 [38]. In contrast, in our study, deficiency in the *C. elegans* homolog of MFN1/2, *fzo-1*, which is required for mitochondrial fusion, did not seem to impact baseline neurodegeneration, at least during the time points included in this study. However, deficiency in the fission protein *drp-1* trended toward increased neurodegeneration by day nine in non-exposed controls (*p* = 0.11) despite the deficiency being generally protective at earlier time points against 6-OHDA exposures. This supports the idea that fission deficiency leading to hyperfusion of mitochondria may be protective due to the buffering effect of hyperfused mitochondria; the damage to mitochondria is diluted. However, if that buffering capacity is exceeded, such as in aging animals, the inability to separate and fragment out damaged mitochondrial content through the process of fission to allow for mitophagy-mediated removal of damage would cause sensitization overall to neurodegeneration.

We previously reported dramatic effects of genetic manipulation of mitochondrial fusion and fission on mitochondrial morphology and function [26]. Functional respiration measurements using the Seahorse Extracellular Flux Analyzer revealed that fusion deficiency in *fzo-1* mutants led to reduced maximal respiration and reduced proton leak, while fission deficient *drp-1* mutants had increased basal respiration, reduced maximal respiration, and a trend toward increased proton leak. This agrees with recent evidence that high mitochondrial fusion promotes a higher proton leak to relieve oxidative stress [39]; there was also a recent report stating that uncoupling is protective against dopaminergic neurodegeneration [40]. We previously reported that *pink-1* and *pdr-1* mutants altered mitochondrial structure and function—both mutations cause a similar degree of hyperfusion of mitochondrial networks that is comparable or even greater than mutants deficient in the fission gene *drp-1.* Furthermore, in addition to similar morphology, both *pink-1* and *pdr-1* had the expected increase in proton leak. Although the degree of increased leak is slightly larger in *pink-1* mutants, it seems unlikely that this explains their resistance to toxicity, since *pdr-1* mutants also had elevated proton leak. We therefore wondered whether the baseline hyperfused mitochondria would have distinct dynamic responses to the toxic exposures; however, we did not see a striking difference in mitochondrial morphology between *pink-1* and *pdr-1* following 6-OHDA exposure despite clear differences in neurodegeneration. A limitation associated with this reasoning is that we analyzed mitochondrial morphology in muscle rather than dopaminergic neuron, cells, and proton leak at a whole-organism level. An alternate explanation is that there is a protective decrease in 6-OHDA uptake in the *pink-1*; Cooper et al. [41] found that *pink-1* loss altered dopaminergic behavior in a fashion consistent with altered dopamine handling, potentially reflecting reduced uptake of dopamine (and thus 6-OHDA). Finally, our model proposes that mitochondrial fission is required for mitophagy, but recent work suggests that this may not always be the case [42].

The mitophagy-related genes *PINK1* and *PRKN* are well-studied in the context of neurodegeneration, and homozygous mutations in each gene in humans promote familial early-onset PD but with varying age-of-onset and incomplete penetrance, suggesting a potential role for environmental factors in disease initiation and progression [4,43]. In this study, there was a robust gene-by-environment interaction between *pink-1* and *pdr-1* mutations and UVC or 6-OHDA exposures. For UVC, both mutations made the animals much more sensitive to neurodegeneration after mtDNA damage caused by UVC exposure. This finding is supported by work in mice showing that *PRKN* knockout mildly accelerates development of Parkinsonian effects in mice engineered to have mtDNA double-stranded breaks in dopaminergic neurons [44] and in Mutator mice with proofreading deficiency in the mtDNA polymerase γ [45]. In contrast to the uniform sensitization of mitophagy mutants to UVC, only *pdr-1* mutants were sensitized to 6-OHDA, while *pink-1* mutants were protected. The fact that *pdr-1* and *pink-1* deficiencies had opposite effects seems surprising given their involvement in the same pathway; however, different effects subsequent to the same stressor have been observed before [30,46]. Indeed, a growing literature demonstrates that different effects for *pdr-1* and *pink-1*, while not universal [37,47], are also not uncommon. For example, *pdr-1* deficiency has been previously associated with increased sensitivity to manganese lethality [48], methyl mercury toxicity [49], and α-synuclein toxicities [47] but was protective against bacterial metabolite-induced dopaminergic neurodegeneration [37]. *pink-1* deficiency protected against rotenone and paraquat toxicities [50] as well as bacterial metabolite-induced dopaminergic neurodegeneration [37] but sensitized against α-synuclein toxicities [47,51]. It should be noted that in most or all such studies, loss of *pink-1* and *pdr-1* led to basal dopaminergic neurodegeneration or other pathologies, such that “protection” could also be interpreted as a failure to exhibit additional stressor-induced dysfunction above that resulting from the background genetic deficiency. For example, while Martinez-Finley et al. [49] observed sensitivity to methylmercury toxicity and decreased dopaminergic behavioral function in *pdr-1* mutants, the same mutants failed to show any change in behavior after exposure to methylmercury, while wild-type showed impaired behavior.

Although the role of mitophagy in removing damaged organelles is important for neuronal function and survival in the face of environmental insults, *pink-1* deficiency may also impact other processes due to its mitophagy-independent functions. Loss of *pink-1* in mice [52] and worms [26] caused impairment of mitochondrial respiration. In particular, there is evidence that *pink-1* deficiency has direct effects on post-translational modification and function of mitochondrial respiratory complex I [53,54]. Furthermore, emerging evidence suggests that the cytosolic pool of *pink-1* can promote cell survival and neuronal differentiation, potentially through mediating mitochondrial motility in the cell [55]. Finally, recent evidence indicates that stress-induced mitochondrial fragmentation can result in daughter mitochondria that vary in form, membrane polarization, and susceptibility to mitophagy [56]. Further characterizing these mitophagy-independent activities of *pink-1* may help explain the differential sensitivities observed herein between *pink-1* and *pdr-1* mutants.

Overall, our results and those of others point to complex gene–environment mitochondrial interactions that may contribute to PD and related conditions associated with dopaminergic neuron loss. Additional genes and toxicants that impact mitochondria appear to be a fruitful area of future inquiry into the etiology of PD. From a regulatory and personal medicine perspective, these potential sensitivities could be of great importance to people who carry homozygous mutations in these genes. For example, mtDNA damage that is difficult to repair would persist without a functional PINK/Parkin mitophagy pathway. Therefore, individuals with deficiencies in mitophagy genes may be particularly vulnerable to exposures to mitochondrial toxicants that cause persistent mtDNA damage. This is of great environmental health relevance, because such damage is induced by common environmental pollutants such as many polycyclic aromatic hydrocarbons, heterocyclic amines, and mycotoxins, as well as some chemotherapeutics such as cisplatin and others [3]. 

## 4. Materials and Methods

### 4.1. C. elegans Culture

Populations of *C. elegans* were maintained on K-agar plates [57] seeded with OP50 *Escherichia coli* at 20 °C until young adulthood, when they were dosed and evaluated. 

Synchronized populations of nematodes were obtained by hypochlorite treatment of gravid adults in order to isolate the eggs [58]. Worms were allowed to hatch for no more than 16 h in K^+^ medium, previously referred to as “complete K-medium” [59]. Worms were transferred by washing off agar plates or treatment wells and rinsing (after centrifugation at 2200 *g* for 2 min) with K medium.

*C. elegans* strains used were BY250 (*vtIs7*[P*_dat-1_*::GFP]), BY200 (*vtIs1*[P*_dat-1_*::GFP, *rol-6* (su1006)]), UA226 (*pink-1*(*tm1779*); *vtIs1*[P*_dat-1_*::GFP, *rol-6* (su1006)]), UA227 (*pdr-1*(*tm598*); *vtIs1*[P*_dat-1_*::GFP, *rol-6* (su1006)]), and CU5991 (*fzo-1*(*tm1133*)), and CU6372 (*drp-1*(t*m1108*)). BY250 was generously provided by Michael Aschner (Vanderbilt University). The *fzo-1* tm1133 strain was provided by Alexander van der Bliek (University of California Los Angeles, Los Angeles, CA, USA), and the *drp-1* tm1108 deletion strain was provided by Ding Xue (University of Colorado Boulder, Boulder, CO, USA). 

Genetic crosses of *fzo-1* and *drp-1* mutant animals with the BY250 *Pdat-1::GFP* transgene were performed by first enriching the male population in the BY250 strain with a 1 h heat shock of L4-stage animals at 35 °C. Their resulting offspring were enriched in males; male BY250 nematodes were then mated with *fzo-1* and *drp-1* hermaphrodites in a ratio of 1 hermaphrodite per 3 males. Homozygous strains were then obtained and verified by PCR genotyping. Genotyping primers used were GCTCTGTCTGCTCACGGTAA (fwd) and CTCATTGCATGTACGGTGTA (rev) for *fzo-1* (419 bp deletion + 14 bp insertion) GAGGTTAAGCCCATGCAATA (fwd) and TGAGTAAGTGGCAGGATCGA (rev) for *drp-1* (tm1108, 425 bp deletion + 18 bp insertion). 

### 4.2. Toxic Exposures

For UVC exposures in *drp-1* and *fzo-1* mutants, synchronized L1 larvae were plated onto agar plates without food or peptone and were dosed three times with 0, 7.5, or 10 J/m2 UVC radiation (Figure 2A). Following exposure, the worms were provided with food and allowed to develop. In *pink-1* and *pdr-1* mutants, young adult nematodes were exposed to UVC (0, 25, 50, or 75 J/m^2^ each day) for three days in a row in unseeded K-agar plates with no peptone (Figure 4A); this allowed for mtDNA to accumulate damage while nDNA was repaired [25]. In between doses, worms were kept on seeded agar plates, as at that age, developmental cell division has ceased and dilution of mtDNA damage is not a concern [60]. 

For *fzo-1* and *drp-1* exposures to 6-OHDA, L4 stage worms were exposed to 50 or 100 mM 6-OHDA (Figure 3A) for 1 h in ascorbic acid (to delay oxidation; ascorbic acid was present in all exposures and controls at a concentration of 10 mM). For *pink-1* and *pdr-1* 6-OHDA exposures, young adult nematodes were exposed to 0, 33, or 100 mM 6-OHDA solution in 10 mM ascorbic acid (Figure 5A). Exposures were done in 1.7 mL tubes (1000 worms per tube) and lasted 1 h. After, worms were washed 4 times in K medium and transferred to seeded K-agar plates. 

### 4.3. Dopaminergic Neurodegeneration Assay

After toxic exposure, worms were sampled at various time points (indicated on individual figures) for evaluation of dopaminergic neuron morphology. Treated *C. elegans* were picked onto a 2% agar pad and immobilized with 15 µl of 156 mM tetramisole hydrochloride (Sigma-Aldrich, St. Louis, MO, USA). Nematodes were examined using a Zeiss Axioskop microscope, and neuronal morphology was assessed by individual observation of each cephalic (CEP) neuron using a modification of the method described in Gonzalez-Hunt et al. 2014 [23]. Neurons were assigned a score from 0 to 2 based on the amount of morphological abnormalities present [23]. The score categories were as follows: 0 = no observable damage; 0.5 = blebs on equal or less than half of dendrite, no breaks; 1 = blebs on more than half of dendrite, no breaks; 1.5 = breaks, equal or more than half of dendrite still present; 2 = breaks, less than half of dendrite still present (includes completely absent dendrite).

Each experiment was repeated three times with 10 worms analyzed per treatment at each time point. All scoring was done with the experimenter blind to strain and treatment.

### 4.4. Targeted Gene Expression Measurements

The mRNA of approximately 1000 worms was extracted using the RNeasy mini kit (QIAGEN, Hilden, Germany) 24 h after 6-OHDA exposure. Worms were washed off plates and allowed to clear their guts for 15 min. They were then transferred to 1 mL RLT buffer plus 10 µL β-mercaptoethanol and flash-frozen in liquid nitrogen. After bringing them to room temperature, zirconia/silica beads were added to the tubes, and samples were homogenized using a bullet blender (Next Advance). The homogenate was transferred to a new 1.7 mL tube and kept at room temperature for 5 min. These tubes were then centrifuged, and the supernatant was transferred to a new microcentrifuge tube. We then followed steps 5–12 of the RNeasy Mini handbook. Once the mRNA was extracted and quantified, it was converted to cDNA using the High-Capacity cDNA Reverse Transcription Kit (Applied Biosystems, Foster City, CA, USA). Gene expression was measured by real-time PCR as described in [61]. Primers used were as follows: *cdc-42* forward-5′-GAG AAA AAT GGG TGC CTG AA-3′, reverse-5′-CTC GAG CAT TCC TGG ATC AT-3′ (111 bp, published in); *ctl-2* forward–ACCCAGAAGCGTAATCCACA reverse–CCTTGAGTTGGCTTGAAATGGA (224 bp); *trx-1* forward–AGCGGAAGATCTTTGTTCCA reverse–AATTGCGTCTCCATTCTTGG (76 bp); *gst-10* forward–TGGGAAGAGTTCATGGCTTG reverse–AACTTCACTAGAGCCTCCGG (172 bp). Annealing temperature was 60 °C for all primers. All samples were run in triplicate, and the experiment was repeated three times.

### 4.5. Mitochondrial Morphology Evaluation

This protocol was adapted from Gonzalez-Hunt et al. 2014 [23]. After exposure to 33 mM 6-OHDA, worms (strains BY200, UA226, and UA227) were kept on OP50 plates for 20 h at 20 °C. They were then washed with K medium and allowed to clear their guts for 15 min. Worms were then transferred to 12-well plates at a density of 1000 worms/well (one well per treatment and strain). Each well had MitoTracker Red CMXRos (Molecular Probes, Eugene, OR, USA) at 3.7 µM, 100 µL of 2× UVC-inactivated *E. coli,* and K medium up to 1 mL. UVC-inactivated *E. coli* was generated via exposure to 1000 J/m^2^ UVC with the UVLMS-38 EL Series Lamp primarily emitting at 254 nm (UVP, LLC) as previously described in Meyer, et al. [62]. Worms were incubated in the dye for 4 h at 20 °C and then washed off with K medium and allowed to clear their guts for 15 min. They were then transferred to a fresh 12-well plate with 100 µL of 2 × UVC-inactivated *E. coli* and K medium up to 1 mL (per well) for imaging.

Confocal imaging was performed immediately after the washes (evaluated with a Zeiss 510 for the first two experiments and a Zeiss 780 upright confocal for the third experimental repetition). Treated *C. elegans* were picked onto a 10% agar pad and immobilized with 15 µl of 156 mM tetramisole hydrochloride (Sigma-Aldrich, St. Louis, MO, USA). A z-stack of the area adjacent to the worm pharynx (between pharyngeal bulbs) was taken at 63 ×, 2.9 zoom, and at 0.5 µm intervals. Three worms per treatment group were imaged (six per strain total). The experiment was repeated three times.

### 4.6. Statistical Analyses

Data were analyzed with JMP Pro for Mac (Version 13.0.0, SAS Institute Inc., Cary, NC, USA). One- or two-factor analysis of variance (ANOVA) was used to evaluate the effect of the toxic exposures on mRNA levels and mitochondrial morphology. For evaluation of UVC or 6-OHDA on neurodegeneration, the Fisher’s exact test (FET) was used to compare the effect of the exposures on each mutant and/or dosed strain with the effect on the control strain. Each dose and time point was assessed independently. A *p*-value of less than 0.05 was considered statistically significant.

## Figures and Tables

**Table 1:** *p*-values (Fisher’s exact test) for strain comparisons (*drp-1* and *fzo-1* vs. wild-type (WT)) after UVC exposure in L1 larval *C. elegans*.

Timepoint	Dose (J/m^2^)	*fzo-1* vs. *WT*	*drp-1* vs. *WT*
48 h	0	0.7991	0.6889
	7.5	N.D. ^1^	0.2973
	10	N.D.	<0.001 *
96 h	0	0.1127	0.1361
	7.5	N.D.	0.0071 *
	10	N.D.	0.3512
9 d	0	0.0237 *	0.1157
	7.5	N.D.	0.8769
	10	N.D.	0.0222 *

^1^ N.D., Not Determined. Neurodegeneration data were not collected for *fzo-1* mutants exposed to UVC because the exposure caused the animals to arrest and die as L1 larvae. *, *p* < 0.05, Fisher’s exact test

**Table 2:** *p*-values (Fisher’s exact test) for strain comparisons (*drp-1* and *fzo-1* vs. WT) after 6-OHDA exposure in L4 *C. elegans*.

Timepoint	Dose (mM)	*fzo-1* vs. *WT*	*drp-1* vs. *WT*
48 h	0	<0.0001 *	0.1740
	50	<0.0001 *	0.0018 *
	100	<0.0001 *	<0.0001 *
6 d	0	0.4128	0.0053 *
	50	<0.0001 *	0.9166
	100	<0.0001 *	0.0040 *

*, *p* < 0.05, Fisher’s exact test.

**Table 3:** *p*-values (Fisher’s exact test) for strain comparisons (*pink-1* and *pdr-1* vs. WT) after UVC exposure in L4 *C. elegans*.

Timepoint	Dose (J/m^2^)	*pdr-1* vs. *WT*	*pink-1* vs. *WT*
24 h	0	0.8599	0.7267
	25	0.0438 *	0.2373
	50	0.0119 *	0.0005 *
	75	<0.0001 *	<0.0001 *
48 h	0	0.0517	0.1928
	25	0.7275	0.4445
	50	0.1419	0.5351
	75	0.3884	0.3332
72 h	0	0.5833	0.7540
	25	0.0124 *	0.9593
	50	0.0002 *	0.9467
	75	0.0001 *	<0.0001 *

*, *p* < 0.05, Fisher’s exact test.

**Table 4:** *p*-values (Fisher’s exact test) for strain comparisons (*pink-1* and *pdr-1* vs. WT) after 6-OHDA exposure in young adult *C. elegans*.

Timepoint	Dose (mM)	*pdr-1* vs. *WT*	*pink-1* vs. *WT*
24 h	0	<0.0001 *	0.6336
	33	<0.0001 *	0.3738
	100	<0.0001 *	<0.0001 *
48 h	0	0.0030 *	0.1894
	33	<0.0001 *	0.0108 *
	100	<0.0001 *	<0.0001 *
72 h	0	0.4511	0.2050
	33	0.0680	<0.0001 *
	100	<0.0001 *	<0.0001 *

*, *p* < 0.05, Fisher’s exact test.

## Abbreviations

mtDNA	mitochondrial DNA
6-OHDA	6-hydroxydopamine
UVC	ultraviolet C radiation
PD	Parkinson’s Disease
